# Discovery of Novel CSF Clearance Mechanism of Idiopathic Normal Pressure Hydrocephalus and Alzheimer's Disease

**DOI:** 10.1002/alz70855_098961

**Published:** 2025-12-23

**Authors:** Anssi Lipponen, Sami Heikkinen, Joel Räsänen, Evan Macosko, Tarja Malm, Mikko Hiltunen, Ville Leinonen

**Affiliations:** ^1^ University of Eastern Finland, Kuopio, Finland; ^2^ University of Eastern Finland, Kuopio, Pohjois‐Savo, Finland; ^3^ Kuopio University Hospital, Kuopio, Finland; ^4^ Broad Institute of MIT and Harvard, Cambridge, MA, USA

## Abstract

**Background:**

In idiopathic normal pressure hydrocephalus (iNPH) and Alzheimer's disease (AD), clearance of cerebrospinal fluid (CSF) is impaired, leading to the buildup of harmful proteins and metabolic waste in the brain, contributing to disease pathology. A significant number of iNPH patients have AD as a comorbidity, and both diseases display impaired memory and altered levels of amyloid beta (Aβ) and microtubule‐associated protein Tau (Tau) in CSF, suggesting intersecting disease mechanisms. Genetic variants may cause loss‐of‐function mutations that affect protein levels or alter gene expression, potentially revealing novel pathogenic or protective mechanisms. We recently conducted a genome‐wide association study (GWAS) for iNPH using FinnGen data and identified the *SLCO1A2, MLLT10, AMZ1/GNA12*, and *C16orf95 loci* (Räsänen et al. 2024).

**Methods:**

To confirm and identify further genetic variants at the four GWAS‐identified loci, we conducted whole genome sequencing (WGS) on ∼300 iNPH patients from the Kuopio iNPH cohort. To test the effects of the discovered variants on phenotype, we associated the genotypes to phenotypes within the Kuopio iNPH cohort, which includes immunohistochemistry from human brain biopsies, CSF biomarkers, and functional and cognitive data from patients in WGS. We will also utilize FinnGen phenotype and genotype data, including patients with NPH and AD diagnoses, to evaluate the variants' effects on onset age and CSF biomarker levels. Finally, we aim to validate the variants' effects on Aβ42 and Tau clearance from CSF to blood in a cell model of the blood‐CSF barrier (BCB).

**Results:**

Our preliminary results indicate that WGS‐confirmed variants in *SLCO1A2* loci affect the onset age of iNPH and AD. Additionally, these variants are associated with altered Aβ42 levels in the CSF of iNPH patients.

**Conclusion:**

Preliminary analysis indicates a functional proof‐of‐concept where the analysis of genotype and phenotype data can be used to identify clearance‐related variants. We aim to further validate the variants' effects on CSF clearance in a model of the BCB.

